# Interactive effects of past and present environments on overwintering success—a reciprocal transplant experiment

**DOI:** 10.1002/ece3.82

**Published:** 2012-05

**Authors:** Tuula A Oksanen, Minna Koivula, Esa Koskela, Tapio Mappes, Carl D Soulsbury

**Affiliations:** 1 Centre of Excellence in Evolutionary Research, Department of Biological and Environmental Science, University of JyväskyläFinland; 2 MTT, Biotechnology and Food Research, Biometrical GeneticsJokioinen, Finland; 3 Department of Biological and Environmental Science, University of JyväskyläFinland

**Keywords:** Delayed density dependence, life history, *Myodes glareolus*, population dynamics, reciprocal transplant experiment

## Abstract

Life-history traits are influenced by environmental factors throughout the lifespan of an individual. The relative importance of past versus present environment on individual fitness, therefore, is a relevant question in populations that face the challenge of temporally varying environment. We studied the interacting effects of past and present density on body mass, condition, and survival in enclosure populations of the bank vole (*Myodes glareolus*) using a reciprocal transplant design. In connection with the cyclic dynamics of natural vole populations, our hypothesis was that individuals born in low-density enclosures would do better overwintering in low-density enclosures than in high-density enclosures and vice versa. Our results show that the effect of summer (past) density was strong especially on survival and body mass. The response of body mass to summer density was negative in both winter (present) density groups, whereas the response of survival probability was nonlinear and differed between the winter density groups. In particular, our data show a trend for higher overwintering success of individuals originating from the lowest summer densities in low winter density and vice versa. We therefore conclude that the capacity of individuals to respond to a change in density was constrained by the delayed density-dependent effects of environment experienced in the past. These effects have the potential to contribute to vole population dynamics. Possible mechanisms mediating the effects of past environment into present performance include both intrinsic and environmental factors.

## Introduction

Conditions during early development, such as environmental or parental effects, can have long-term consequences on individual life histories (Beckerman et al. 2002). Moreover, many species are capable of developing rapid responses to changes in their immediate environment such as bursts of compensatory growth (Metcalfe and Monaghan 2001). Variation in individual life histories may therefore be considered an outcome of the interaction between genes and environmental factors but also an interaction between environmental factors operating during the different phases of the life cycle (Monaghan 2008). This idea was first adopted by researchers focusing on compensatory growth, that is accelerated growth after a period of restricted development that enables individuals to catch up with/to their nonrestricted conspecifics (Hornick et al. 2000; Metcalfe and Monaghan 2001, 2003). The importance of compensatory growth on individual life histories has been widely examined, for example, in relation to the length of lifespan (Ozanne and Hales 2004; Inness and Metcalfe 2008), dominance status (Royle et al. 2005), and metabolism (Criscuolo et al. 2008). In a more general ecological context, the life-history effects of changes in food quality have been demonstrated by several food manipulation studies (e.g., Plaistow et al. 2006; Taborsky 2006; Barrett et al. 2009; Helle et al. in press).

Food resources and diet quality are environmental factors that typically vary across the lifespan of an individual, and are therefore biologically meaningful targets for the research on delayed life-history effects. Additionally, another potentially important variable in this context is population density (Beckerman et al. 2002). For many species, including small mammals, the life-history effects of population density are well documented and considered to be evolutionarily significant. For example, high population density is known to suppress sexual maturation (Prevot–Julliard et al. 1999; Ergon et al. 2001a), decrease reproductive success (Koskela et al. 1999), and reduce the growth rate of juveniles (Ostfeld and Canham 1995). In cyclical populations, there is commonly observed pattern known as the Chitty effect (Chitty 1967; Boonstra and Krebs 1979), in which individuals are larger during the increase and peak phase of fluctuations than in the declining and low phase. In the context of predictable density fluctuations, it has even been suggested that females may modify the phenotype of their offspring according to the density of the population to improve their fitness (Lacey 1998). This could be achieved through adaptive maternal effects, that is, an adaptive relationship between offspring phenotype and the environment experienced by the mother (Rossiter 1996; Mousseau and Fox 1998; Marshall and Uller 2007).

At the individual level, density-induced changes in the life-history traits of small mammals are most likely based on phenotypic plasticity that enables quick responses to changes in the environment (Agrell et al. 1995; Norrdahl and Korpimäki 2002). For example, Ergon et al. (2001b) conducted an extensive field transplant experiment by moving field voles (*Microtus agrestis* L.) between sites that differed in average overwintering body mass. Their results showed that transplanted voles did not retain the characteristics of their source population and that the immediate environment therefore had an overriding role in shaping the body size. Moreover, as reproduction started earlier in sites with higher body mass, adjusting body size to the immediate environment carried a clear benefit by enabling reproduction concurrently with the rest of the population and contributing to the population growth rate. However, according to our knowledge, there have been no previous experiments on small mammals using a reciprocal transplant design that has manipulated density.

**Figure 1:**
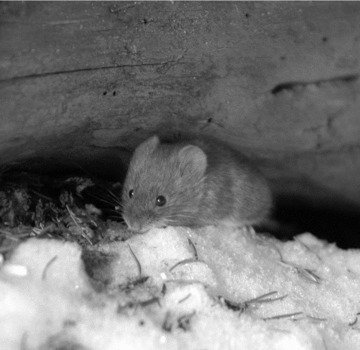
Study species, the bank vole *Myodes glareolus* is a small rodent species common in northern Europe. The main habitats are forests and fields, and the diet typically consists of forbs, shoots, seed, berries, and fungi. Photo credit: Matti Laine.

We aimed to study the effects of past and present densities on the body mass, condition, and survival of young bank voles (*Myodes glareolus* Schreber) (Fig. 1), by designing an experiment where individuals born in an enclosure population of 8, 12, 16, 20, or 24 adult individuals were transplanted in either a low (9–10 adult individuals) or a high (18 adult individuals) density enclosure to overwinter. We hypothesized that a change in population density would lead to a mismatch between an immediate adaptive response and future environment, and thereby to lowered individual fitness (Bateson et al. 2004). This idea parallels the concept of predictive adaptive responses (PAR) of human evolution, which states that organisms preset their physiology according to the cues of their prenatal environment in expectation that particular physiology will match their future environment (Gluckman et al. 2005). In our study, however, we cannot quantify how the population density experienced by the mothers translated into the intrauterine environment experienced by the study animals.

In vole populations, density and density-related factors (e.g., availability of food resources and free breeding territories) have been found to affect several life-history traits such as maturation, reproductive success, and susceptibility to the costs of reproduction (Bujalska 1985; Koskela et al. 1999; Prevot–Julliard et al. 1999; Oksanen et al. 2007; Mappes et al. 2008). Population density is therefore likely to have a substantial effect on the development of young individuals. It was predicted that the summer densities experienced by individuals during early development would influence their life-history trajectories, and that the reciprocal transplant design would challenge the individuals to respond to the changed conditions within the limits of their capacity to further phenotypic plasticity. More specifically, we tested if individuals born in low summer densities would do better overwintering in low winter density than in high winter density and vice versa. This is meaningful in the context of vole population cycles because if individuals that are born during the peak phase of fluctuations have relatively low fitness during the declining phase, it could speedup the decline in population density and so amplify the fluctuations.

## Materials and Methods

### Study site

Experimental animals were young bank voles raised in enclosure colonies at Konnevesi Research Station. Colonies originated from laboratory-born descendants of wild individuals originally captured at our study site (Konnevesi, central Finland 62°37′N, 26°20′E). There was density variation in the enclosures (8, 12, 16, 20, or 24 adult individuals per 0.2-ha enclosure) that had been established for an earlier enclosure experiment (see Oksanen et al. 2007). These densities were all relatively high and correspond to the peak phase of the multiannual population cycles of natural bank vole populations (Yoccoz et al. 2001). However, due to unavoidable natural mortality the density of eight individuals was considered a suitable starting point for the low-density treatment. Density of 24 individuals (120 individuals/ha) corresponds to a very high but not unusual density in natural populations. These densities, that is the densities prevailing when the experimental animals were born are referred to as summer densities. Sex ratio of adult individuals in the enclosures was 1:1.

The 11 0.2-ha study enclosures were situated in an old field. To monitor the animals, 20 multiple-capture live traps were distributed in each enclosure in a 5 × 4 grid with a distance of 10 m between traps. Each trap was covered with a galvanized sheet metal chimney that reduced exposure to precipitation and temperature extremes. Enclosure fences were constructed of 1.25-m high galvanized sheet metal that was embedded 0.5 m into the ground. The fences were high enough to enclose the study populations, but did not prevent predation by mammalian and avian predators. The voles were dependent on naturally occurring food resources except during trapping periods, when the traps were baited with oats and sunflower seeds.

### Reciprocal transplant experiment

Following the end of the breeding season in October, all individuals (adult individuals and the offspring born during the breeding season) were trapped from the enclosures and transferred to the laboratory. Offspring from the first litter of each adult female were measured with an electronic scale for their body mass (to nearest 0.01 g) and with a digital caliper for their head width (to nearest 0.1 mm) and released back into six enclosures in two different densities to overwinter. In total, this reciprocal transplant experiment included 73 individuals released at ca. 91 days of age (42 males and 31 females). Four of the winter enclosures had low density (nine or 10 individuals) and two winter enclosures had high density (18 individuals). These densities are referred to as the winter densities. Ratios of males and females in each enclosure were adjusted as close to 1:1 as possible. Approximately one-half of the individuals originating from each summer density class (8, 12, 16, 20, or 24) were assigned to low winter density enclosures and the other half was assigned to high winter density enclosures. Individuals originating from the same litter (24 litters in total) were divided into different treatments and replicate enclosures to randomize the effects of common origin and to avoid inbreeding. The birth of these individuals had been monitored in laboratory during an earlier experiment by Oksanen et al. (2007), and the birth date, mother, and number of siblings of these individuals were therefore known. The females and their litters were released back into the enclosure, the day following the birth of the pups and the total time the female spent in the laboratory was on average four to five days.

Study design is described in Table 1. Individuals assigned to the high and low winter density groups did not differ in their age, body mass at autumn, or the size of the litter in which they had been reared (mean age [days] ± SE, high: 91.59 ± 0.84, low: 91.39 ± 1.27, Independent samples *t*-test: *t*= 0.136, *n*= 73, *P* > 0.8; mean body mass [g] ± SE, low: 16.8 ± 0.2, high: 16.9 ± 0.2, Independent samples *t*-test: *t*=–0.219, *n*= 73, *P* > 0.8; mean litter size ± SE, low: 5.4 ± 0.2, high: 5.3 ± 0.2, Independent samples *t*-test: *t*= 0.200, *n*= 73, *P* > 0.8). Reproductive history of these individuals was not recorded during the breeding season; however, based on the birth dates, each individual had had a possibility to reproduce at least 1–2 times before the breeding season was over. The animals were trapped again in spring (March) before the next breeding season started and transferred back to the laboratory where their body mass and head width was measured. Condition was estimated as a standardized residual from a linear regression of body mass on head width (Schulte–Hostedde et al. 2005). Individuals that were not caught after an intensive trapping effort were recorded dead. The protocol and the procedures employed were reviewed and approved by the experimental animal committee of the University of Jyväskylä.

**Table 1:** Number of individuals reciprocally transplanted from summer densities to winter densities. Summer densities are 8, 12, 16, 20, and 24 individuals per enclosure in the parental generation and winter densities are either 9–10 (low) or 18 (high) experimental individuals per enclosure.

Summer density past		Winter density	
Enclosure	Density	Low	High
1	8	2	3
7	8	2	2
11	8	8	8
2	12	2	1
9	12	6	6
3	16	2	2
10	16	3	3
4	20	0	0
5	20	3	3
6	24	4	3
8	24	5	5
Total	168	37	36

### Data analysis

**Figure 2:**
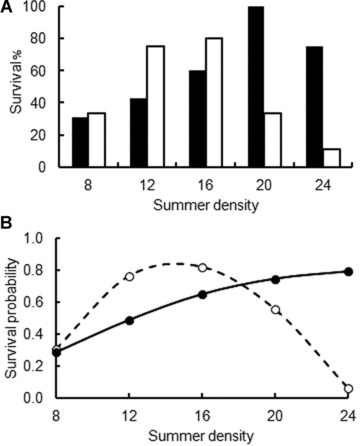
Effects of summer and winter density on survival. (A) The proportion of individuals that survived at low (open bars) and high (filled bars) winter density and (B) the predicted probability of survival over the winter. Open circles and dashed line, low winter density; filled circles and solid line, high winter density. Equation for the curve of the low-density group is y =–0.0099x^2^+ 0.2989x – 1.4345 and equation for the curve of the high density group is y =–0.0017x^2^+ 0.0853x – 0.2883.

Data were analyzed using linear (LMM) or generalized (GLMM) linear-mixed effects models (Bolker et al. 2009), using the lme4 (Bates and Maechler 2009) package in R (R Development Core Team 2009). Sex (female/male) and winter density (low/high) were used as fixed factors and summer density and litter size as continuous covariates. The probability of surviving to the end of the experiment (spring) was analyzed with GLMM with binomial distribution and logit link function. Survival did not fit a linear response with summer density (see Fig. 2A), so we used a quadratic term (summer density^2^) to evaluate if survival varied nonlinearly with summer density. Models including the quadratic term were significantly better than without it (LR test: χ^2^= 9.67, *P*= 0.008). We started from a model that included all relevant factors as main effects and their two-way interactions. The model was then hierarchically simplified by removing the interactions and main effects with nonsignificant *P*-values. However, in the analyses of log transformed body mass and condition, we decided to include the effects of sex and sex-winter density interaction, because they are biologically meaningful due to the sexual size dimorphism observed in the study species (Koskela et al. 2009).

Models outputs were examined for homogeneity and normality of residuals. *P-*values for Gaussian error distributions were estimated by comparison to a probability distribution obtained by 10,000 Markov chain Monte Carlo simulations (Baayen 2007). For all variables, we report effect sizes and 95% confidence intervals (CI) (Nakagawa and Cuthill 2007). In the analysis of data collected in October, the unit for sample size is a litter (*n*= 24 for transplanted litters or 57 for all litters born during the summer) and in the analysis of data collected in March, the unit for sample size is an individual (*n*= 35 [body mass and condition] or 73 [survival]).

## Results

Body mass of the transplanted individuals before the reciprocal transplant procedure had a statistically significant negative relationship with summer density and litter size (litter mean of body mass [g] ± SE: 16.8 ± 0.2, individual range: 13.7–20.5; LMM 10 enclosures, 24 litters: summer density: *t*=–2.31, *P*= 0.038, *r*=–0.52, 95% CI =–0.75/–0.06; litter size: *t*=–3.67, *P*= 0.002, *r*=–0.70, 95% CI =–0.84/–0.35; random effect of study enclosure: <0.001 ± <0.001. The strong influence of density on litter size was demonstrated by a statistically significant negative correlation between summer density and litter size over all 57 litters whose birth had been observed in the laboratory (Spearman's rho =–0.468, *n*= 57, *P*≤ 0.001). Condition, however, was clearly not related to either of these covariates (range: –1.9 to 2.88; LMM: 10 enclosures, 24 individuals, summer density: *t*=–0.704, *P*= 0.480, *r*=–0.18, 95% CI =–0.57/0.32; litter size: *t*=–1.272, *P*= 0.232, *r*=–0.32, 95% CI =–0.65/0.19; random effect of study enclosure: 0.248 ± 0.498).

**Figure 3:**
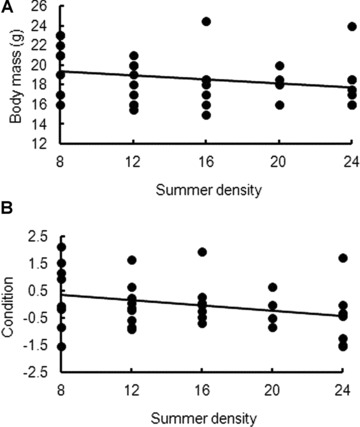
Effect of summer density on body mass and condition. A) Body mass in spring and (B) body condition in spring. Figures are produced from the raw data and the curves fitted are linear.

Forty-eight percent of the transplanted individuals survived over the winter (from October to March). Survival was significantly affected by summer density (Table 2). The response of survival to summer density varied nonlinearly in both winter density groups (Table 2, Figs. 2A, B). Though the interaction term between summer and winter densities was only close to significant (Table 2), it strongly suggested that the distribution of survival probabilities of individuals born in high densities was higher at high winter densities, while the converse was true for low summer densities (Figs. 2A, B). The responses of body mass and condition to summer density were linear and negative in both winter density groups (Figs. 3A, B). The response of body mass was statistically significant, whereas the response of condition was weaker and only marginally significant (Table 3). Interaction terms between summer and winter densities in these models were not significant and only suggested a weak trend for an interactive effect in body mass (Table 3).

**Table 2:** Probability of survival. Binomial GLMM with summer density (SD) and winter density (WD) as independent variables (*n*= 73). The intercept corresponds to an individual in low winter density. Random effect of study enclosure is included into the model (estimated parameter for variance component ± SD: 0.297 ± 0.545). Effect size (*r*) and noncentral 95% confidence intervals (CI) are shown for each variable. Conventions for effect sizes: small effect, *r*= 0.10, medium effect, *r*= 0.30, large effect, *r*= 0.50 (Cohen 1988).

	Estimate	SE	*z*	*P*	*r*	CI
Intercept	−8.543	3.305	–	–		
WD	4.988	4.512	1.11	0.269	0.11	−0.09/0.30
SD	1.343	0.490	2.74	0.006	0.27	0.07/0.44
SD (quadratic)	−0.045	0.016	−2.84	0.004	−0.28	−0.09/–0.45
WD × SD	−0.964	0.658	−1.46	0.143	0.15	−0.05/0.34
WD × SD (quadratic)	−0.038	0.021	1.81	0.070	0.19	−0.02/0.37

**Table 3:** Individual body mass and condition in spring. LMM outputs for the effects of sex, summer density (SD), and winter density (WD) (*n*= 35). Intercept corresponds to a female in low winter density. Random effect of study enclosure is included into the models (estimated parameter for variance component ± SD: 0.004 ± 0.061, 0.088 ± 0.297, respectively). Effect size (*r*) and noncentral 95% confidence intervals (CI) are shown for each variable. Conventions for effect sizes: small effect, *r*= 0.10, medium effect, *r*= 0.30, large effect, *r*= 0.50 (Cohen 1988).

	Fixed effects	Estimate	SE	*t*	*P*	*r*	CI
Body mass	Intercept	3.021	0.145	–	–		
	Sex	0.048	0.044	0.63	0.669	0.10	−0.21/0.39
	WD	−0.322	0.145	−1.39	0.113	−0.22	−0.48/0.10
	SD	−0.0113	0.007	1.85	0.047	0.29	−0.02/0.53
	Sex × WD	0.082	0.094	0.87	0.315	0.14	−0.18/0.42
	WD × SD	0.010	0.009	1.53	0.094	0.24	−0.07/0.50
Condition	Intercept	0.628	1.348	–	–		
	Sex	0.523	0.518	1.01	0.331	0.17	−0.17/0.46
	WD	−2.472	1.543	−1.60	0.120	−0.27	−0.53/0.07
	SD	−0.094	0.050	−1.88	0.065	0.31	−0.02/0.56
	Sex × WD	0.546	0.642	0.85	0.403	0.15	−0.19/0.44
	WD × SD	−0.085	0.058	1.46	0.152	0.25	−0.09/0.51

## Discussion

We conducted a simple enclosure experiment where bank voles raised in varying population densities were transplanted either in low or high density to overwinter. Our aim was to study the interaction between summer and winter density on phenotypic variation in body mass, condition, and survival. The relative importance of past and present environment on individual fitness has previously been studied mainly by manipulating food resources while, according to our knowledge, this was the first experiment utilizing a density-manipulation approach.

Bank vole populations in Fennoscandia show both seasonal and multiannual cyclic fluctuations in population density (Kallio et al. 2009), and individual voles, therefore, frequently face density-related changes in their environment (Hansson and Henttonen 1985; Korpimäki et al. 2005). The actual density fluctuations, however, cannot be easily integrated into experimental designs, and previous studies on density-dependent effects on individual characteristics have mostly relied on correlative data (Tkadlec and Zejda 1998; Norrdahl and Korpimäki 2002) and comparisons between populations with different densities either in natural (Prevot–Julliard et al. 1999; Ergon et al. 2001a) or in seminatural environments (Ostfeld et al. 1993; Ostfeld and Canham 1995; Koskela et al. 1999). Although our design either does not include density fluctuations comparable to natural vole cycles, it indicates the potential of individuals to respond to changes in population density, which is important in the context of delayed life-history effects (Beckerman et al. 2002). Delayed life-history effects are potentially a key mechanism in linking environmental conditions to population level responses such as maternally mediated density effects to the vole cycles (Inchausti et al. 1998). We therefore formulated our hypothesis in the context of vole population cycles, and predicted that individuals born in low densities would do better overwintering in low-density than in high-density environment and vice versa. This could contribute to the cycles, for example, by speeding up the decline phase of the cycle.

According to our results, the effect of summer density was strong on most of the measured variables. Summer density had a negative effect on the size of the litters from which the experimental individuals originated and on the body mass of these individuals before the reciprocal transplant. Moreover, summer density induced a nonlinear response in the probability of survival and had a negative effect on the body mass after the overwintering period (Tables 2, 3 and Figs. 2, 3A). The raw data suggest that the overwintering success of individuals originating from the lowest summer densities was better in low winter density than in high winter density and vice versa (Fig. 2A). The interpretation based on our best fitting model (Table 1, Fig. 2B), however, is not as straightforward. The interactive effects of summer and winter densities on the probability of overwintering survival suggest that the response of the survival probability to the summer density was different between the low and high winter density groups. The response in the low winter density group was clearly quadratic with a peak survival probability at a summer density of approximately 16 (Fig. 2B). Interpreting the results from the point of view of the high winter density group is more complicated as appears that our summer density series was not long enough to catch the full shape of the curve or the peak value of the predicted probability of survival (Fig. 2B). Tendency for relatively low survival in low density may be explained by the possible benefits of increased social tolerance during the nonreproductive season that has been observed in several vole species. For example, voles are less territorial, less active, and share nests and hoards of food (e.g., Webster and Brooks 1981; Wolff and Lidicker Jr 1981; Eccard et al. 2011). In high-density conditions, these behaviors may translate into more effective thermoregulation and co-operative defense against other species. The overall winter survival rate (48%) was similar to a survival rate from an earlier study (51%) conducted in the same study enclosures (Oksanen et al. 2001), suggesting that nothing exceptional happened, for example, in the weather conditions during the winter.

The negative effect of litter size on body mass before overwintering (i.e., age of ca. 91 days) was expected as a trade-off between bank vole litter size and offspring growth and body size has been confirmed in numerous earlier studies (Mappes et al. 1995; Koskela 1998; Oksanen et al. 2001, 2002; Mappes and Koskela 2004). However, as litter size was negatively related to population density as well, in theory, the effect of population density on body mass could have been positive. The negative effect of summer density on body mass therefore suggested that density effects were not mediated by litter size exclusively, but that density had more direct effects on body mass as well.

Compared to previous studies with similar designs, our results only weakly support the findings of the field vole transplant experiment (Ergon et al. 2001b), which suggested an overriding role of the immediate environment in shaping life-history traits and thereby enabled discarding intrinsic mechanisms as an explanation for variation in them. Recently, an overriding effect of an immediate environment has been reproterd in another vole transplant experiment as well. Helle et al. (in press) studied the long-term effects of juvenile and adult environments manipulated by food suplementation in enclosure populations of the bank vole. Their study showed that the reproductive success of females was determined by the quality of the adult environmet whereas the survival of males and the characteristics of the litters were determined by the juvenile environment. In our experiment, the immediate environment seemed to challenge the effect of past environment only in the terms of overwintering survival as demonstrated by the shift in the distribution of the predicted survival probabilities (Fig. 2B). In nonmammalian study systems, Taborsky (2006) and Barrett et al. (2009) studied the interacting effects of dietary conditions during different developmental periods on the life-history traits in cichlids and cockroaches, respectively. Both of these studies conclude that the life-history traits measured were influenced by juvenile growth conditions rather than by resource availability later in life. The results of the current study support their conclusions with the exception of the weak interaction between the past and the present densities in body mass. These rather contradictory results suggest that the relative significance of past versus present conditions may vary between study species as well as traits, mechanisms, and conditions under examination. Building up a general understanding about delayed life-history effects, therefore, will not be an easily attainable goal.

The strongest support to our results comes from an invertebrate system (Plaistow et al. 2006). A food manipulation experiment in soil mites showed that the intergenerational effects of parental nutritional conditions can be context dependent and have complex effects on population dynamics (Plaistow et al. 2006). Furthermore, the persistence and significance of the effects of parental food environment (low, medium, or high) varied between the food environments of the descendants, and that the effects of parental environments were most pronounced when the descendant environment was not restricting, that is, in high food conditions (Plaistow et al. 2006). This result is comparable to our results on survival probability, which suggested that the effect of summer density on survival probability differed between the winter density groups and that the response was more distinct when there was less competition over resources, that is when the winter density was low.

Our data does have some shortcomings; in particular, due to the limited number or individuals available for the reciprocal transplant experiment, it was not possible to include the covariance among individuals born into the same litter or the covariance among individuals originating from the same enclosure in the statistical models. Moreover, it is unavoidable that individuals available for the transplant represent a nonrandom subset of the offspring born during the summer as viability selection occurs over the breeding season. However, our results on body mass and the probability of survival generally seem to support the hypothesis that the life-history trajectories of bank voles are influenced by population density experienced during early development. Furthermore, our results on survival probability suggest that a large-scale change in population density has a potential to influence individual fitness. The results therefore suggest that delayed density-dependent effects may indeed play a role in the cyclic dynamics of vole populations. In enclosure environment, possible mechanisms mediating the effects of maternal environment into offspring performance include maternal effects such as litter size-related factors, maternally derived immunity, and early programming of individual metabolism. However, purely environmental effects such as predation or environmental pathogens cannot be ruled out either.

Moreover, the results suggest that in predictable environments, it would be possible for individuals to improve their fitness by adjusting their reproductive effort (i.e., litter size) to the forthcoming density conditions (Gluckman et al. 2005). However, in unpredictable environments, the capacity of individuals to respond to changes in population density may be constrained by the delayed density-dependent effects of the past environment. In bank voles, this is likely to increase the complexity of reasons leading to cyclic population dynamics and the difficulties faced in explaining them. The results, however, are not only interesting in the context of vole population dynamics and the possible intrinsic and extrinsic causes contributing to them, but also in considering other species including humans, which face the challenge of temporally varying environment, and thereby a possible mismatch between immediate adaptive responses and future environment (Bateson et al. 2004; Gluckman et al. 2005; Rickard and Lummaa 2007; von Bonsdorff et al. 2011).
